# Differential width discrimination task for active and passive tactile discrimination in humans

**DOI:** 10.1016/j.mex.2020.100852

**Published:** 2020-03-19

**Authors:** André Perrotta, Carla Pais-Vieira, Mehrab K. Allahdad, Estela Bicho, Miguel Pais-Vieira

**Affiliations:** a Centro de Investigação em Ciência e Tecnologia das Artes (CITAR), Escola da Artes, Universidade Católica Portuguesa, Porto, Portugal; b Centro de Investigação Interdisciplinar em Saúde-Porto, Instituto de Ciências da Saúde, Universidade Católica Portuguesa, Porto, Portugal; c Centro Algoritmi, Department of Industrial Electronics, University of Minho, Campus Azurem, Guimarães, Braga, Portugal; d Life and Health Sciences Research Institute (ICVS), School of Medicine, University of Minho, Braga 4710-057, Portugal; e iBiMED – Institute of Biomedicine, Department of Medical Sciences, University of Aveiro, Aveiro, Portugal

**Keywords:** Somatosensory cortex, Behavioral task, Finger

## Abstract

The neurophysiological basis of width discrimination has been extensively studied in rodents and has shown that active and passive tactile discrimination engage fundamentally different neural networks. Although previous studies have analyzed active and passive tactile processing in humans, little is known about the neurophysiological basis of width discrimination in humans. Here we present a width discrimination task for humans that reproduces the main features of the width discrimination task previously developed for rodents. The task required subjects to actively or passively sample two movable bars forming a “narrow” or “wide” aperture. Subjects were then required to press one of two buttons to indicate if the bar width was “narrow” or “wide”. Behavioral testing showed that subjects were capable of discriminating between wide or narrow apertures up to distances of 0.1 cm. Electroencephalography (EEG) recordings further suggested distinct topographic maps for active and passive versions of the task during the period associated with the aperture discrimination. These results indicate that the Human Differential Width Discrimination Task is a valuable tool to describe the behavioral characteristics and neurophysiological basis of tactile processing.

• *Active and passive width discrimination has been extensively studied in rodents but not in humans.*
• *Human subjects were capable of discriminating aperture widths of 0.1* cm.
• *Electroencephalography recordings showed that active and passive versions of the task were associated with different topographic maps.*

## Graphical abstract

Representational diagram of the behavioral width discrimination task for active and passive tactile discrimination in humans. (**A)** The customized Graphical User Interface (GUI) allows the experimenter control over a variety of system management functions, such as, running different versions of the width discrimination task, configuring setups, visualizing finger and saving behavioral data. (**B)** The Behavioral Apparatus is where the subjects perform the task. It is composed of a box with a small opening that allows the subject to insert the index finger and perform the width discrimination. Inside the small opening is a variable-aperture width determined by two bars. This variable-aperture width is set by a programmable integrated circuit using an Arduino microcontroller. The frontal panel has a central light, two push buttons on the top right corner, and a four-digit LED display on the left corner. **C)** Steps of Behavioral Protocol: at the start of each trial, the center light is red and the bars are set to a predetermined position, while the subject keeps the finger out of the behavioral apparatus **(1).** When the center light turns yellow, the subject must insert the finger in the opening and wait for the algorithm to detect the correct finger positioning **(2)** as the finger reaches the aperture width, the center light turns green. At this point, if the subject is performing the active version of the task, the subject is allowed to move the finger in order to sample the aperture width. Otherwise, if the subject is performing the passive version of the task, the subject is required to remain with the finger in place, and the bars will move towards the finger stopping at the specified aperture width. In active and passive versions the aperture width can be “narrow” or “wide”. This is the Discrimination period **(3)**. After tactile sampling, the light will turn red and the subject is required to remove the finger from the aperture width **(4)**, after which the subject must press one of two buttons, to indicate if the aperture width was narrow (and the subject should press the Blue button) or wide (and the subject should press the Yellow button) **(5).**

**Figure:**
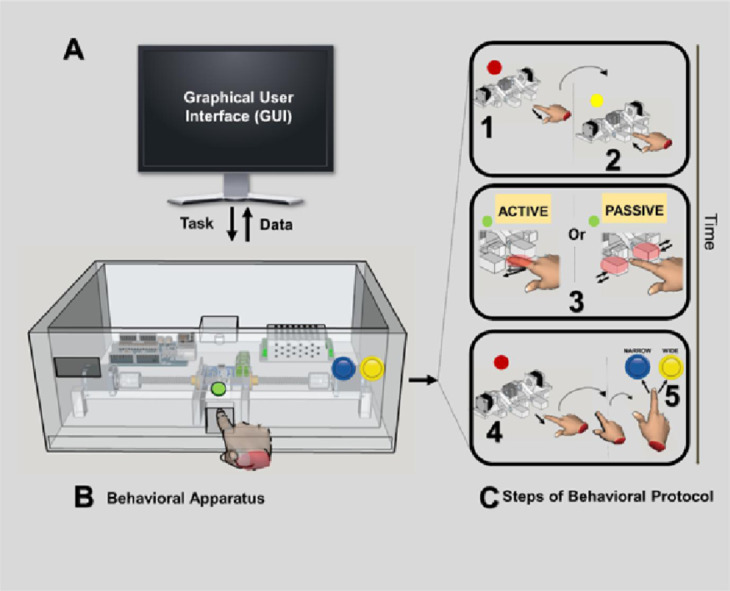

Specifications Table

**Table:** 

Subject Area:	Neuroscience
More specific subject area:	Somatosensory processing
Method name:	Differential width discrimination task for humans
Name and reference of original method:	*(Note: the original task was described for rodents)* Krupa, D.J., Matell, M.S., Brisben, A.J., Oliveira, L.M. and Nicolelis, M.A., 2001. Behavioral properties of the trigeminal somatosensory system in rats performing whisker-dependent tactile discriminations. *Journal of Neuroscience*, *21*(15), pp.5752–5763.
Resource availability:	https://github.com/avperrotta/tactileFeedback

## Method details

### Behavioral apparatus and technical details

**Fig. 1:**
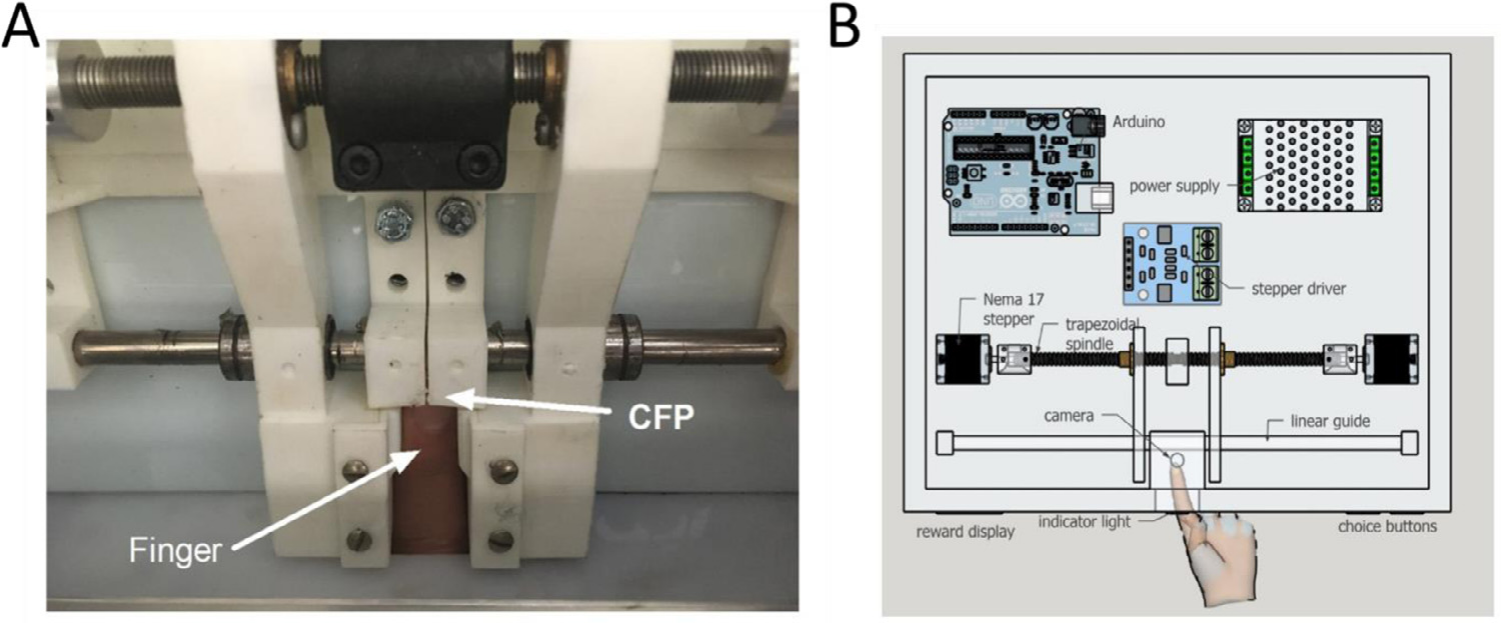
Behavioral apparatus. (A) Overall scheme of behavioral apparatus showing the power supply, Arduino, stepper driver, stepper motor, trapezoidal spindle and camera. On the bottom, is shown the hand of the subject crossing the frontal panel (also see text for details of the hand cover in the frontal panel). (B) Detail of the center-finger place (CFP). View from the top of the behavioral apparatus after removal of top cover.

**Fig. 2:**
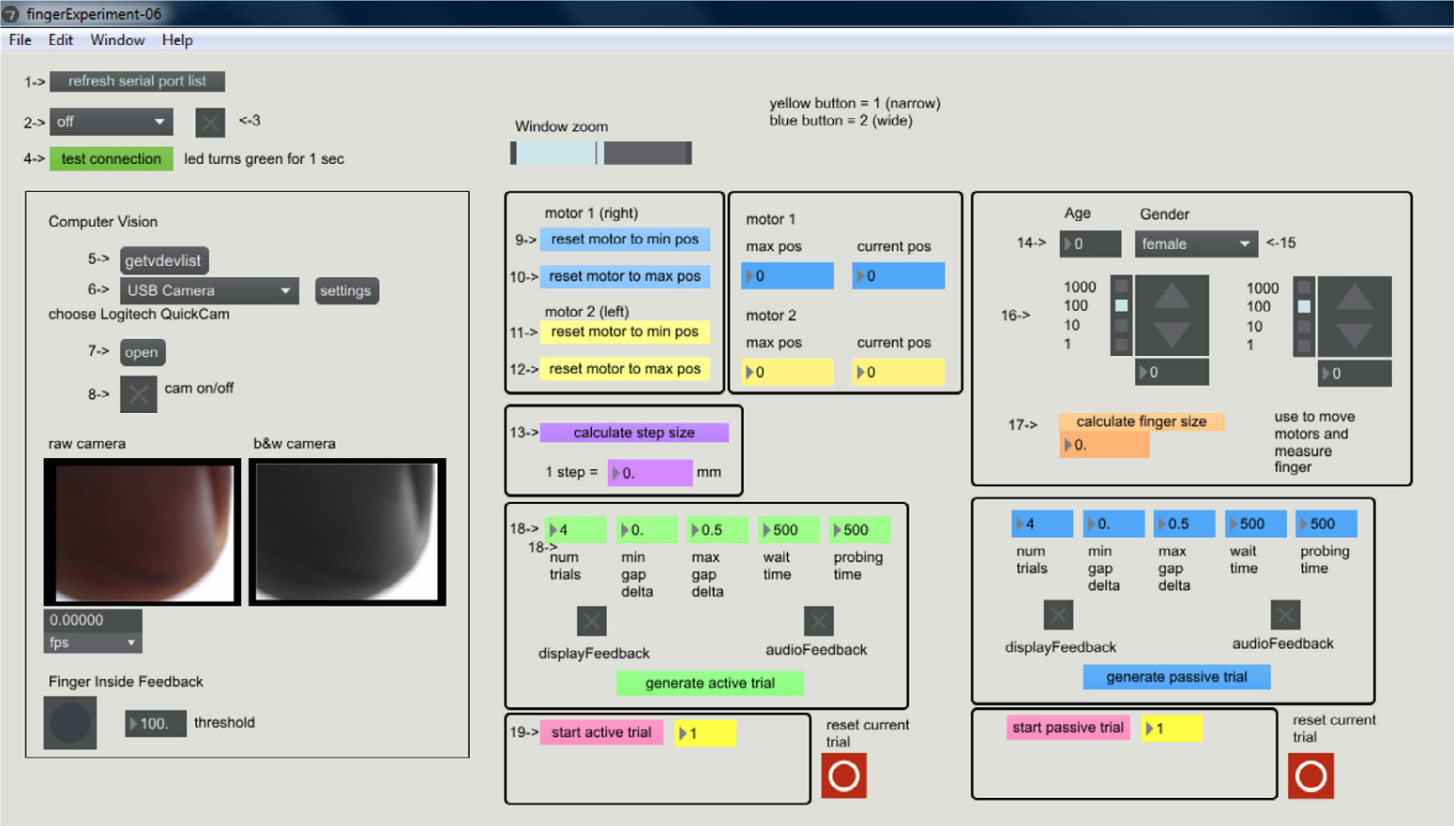
Graphical User Interface (GUI) to control the task.  Options outside the panels (1–4) ensure appropriate communication between the computer and the Arduino as well as Window zoom. Seven main panels are shown: Computer vision (numbers 5–8) motor calibration and subject details (panels with numbers 9–17), session details (number 18), and lastly, starting the session (number 19).

The width discrimination task apparatus hardware is presented in Fig. 1, and the GUI used to control the hardware is presented in Fig. 2. The tactile discrimination experimental apparatus consists of a custom developed electromechanical device controlled by a custom designed computer software that allows for tactile discrimination control in a consistent and repeatable manner.

**Fig. 3:**
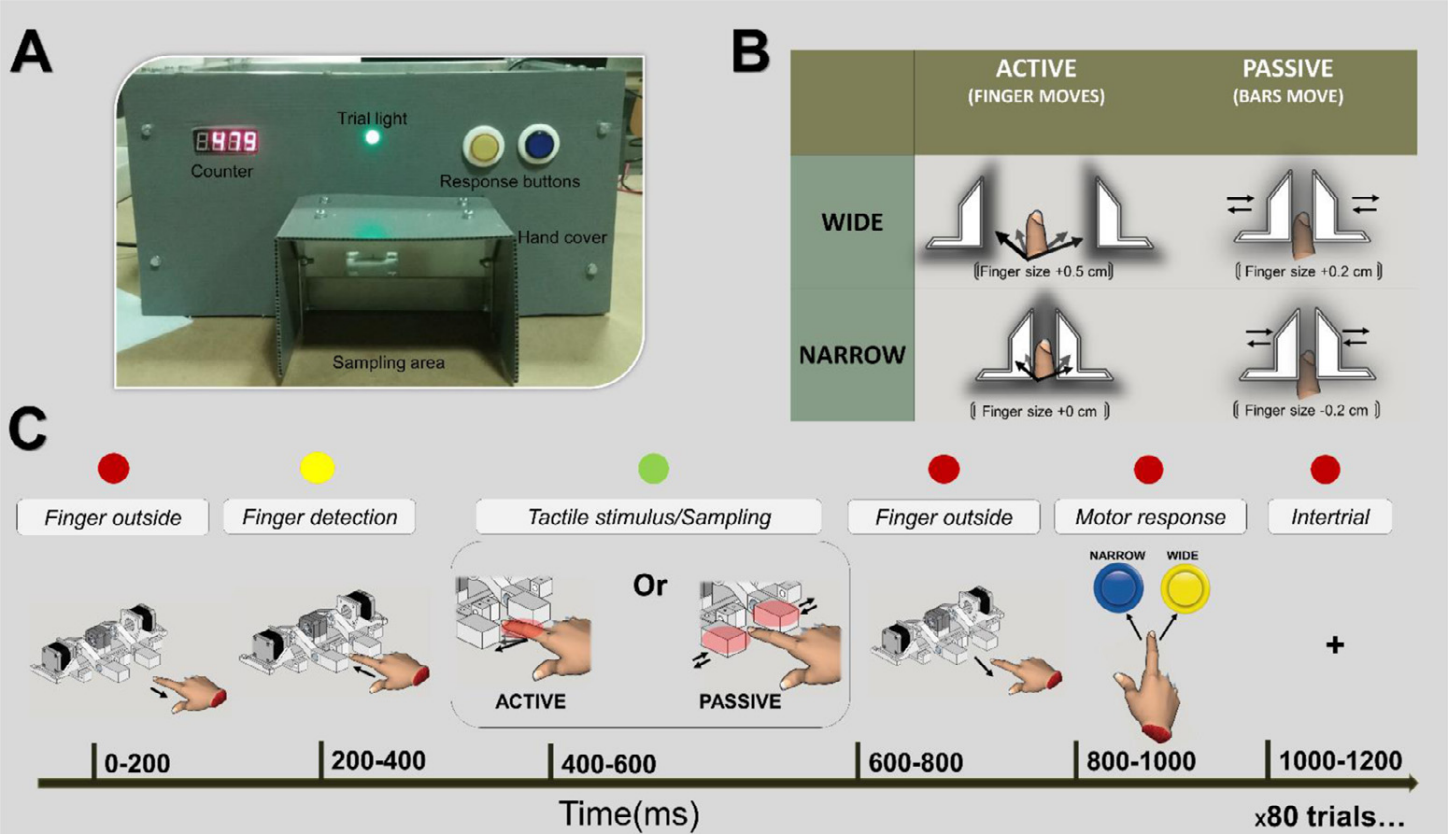
Behavioral protocol. (A) Behavioral box showing the sampling area inside the hand cover. (B) Wide and narrow stimuli can be delivered in active and passive versions of the task. In the figure we show aperture widths that facilitate behavioral performance in each version of the task. (C) When the center light is red the subject needs to keep the finger outside the sampling area. When the light turns yellow, the subject must place the finger inside the sampling area and in the CFP. As the algorithm detects the finger in the correct position the light will turn green and the subject has to either wait for the bars to move (tactile stimulus delivered in the passive version of the task), or the subject is required to explore the aperture width (in the active version of the task). The light will turn red and the subject is required to remove the finger and make a motor response. One of two buttons must be pressed to indicate if the aperture width was narrow or wide. Lastly, the light will remain red, but the algorithm will wait for a preset duration of time before a new trial starts. Note that participants will sometimes spend much longer than 200 ms on a particular portion of a trial.

The apparatus box has the dimensions size of 40 × 35 × 20 cm (length, width, height), with an opening located in the center of the frontal panel. This opening is where the subject's finger is inserted.  An additional hand cover with 10 × 10 × 10 cm (length, width, height; see Fig. 3) prevents the subject from visualizing the aperture width formed by the two bars. The box structure was constructed with an aluminum frame, acrylic bed and plastic and soundproof walls to minimize the aural noise generated by the motors.

The electromechanical part consists of two Nema 17-size stepper motors controlled by an Arduino Mega 2560 and a Dual Bipolar Stepper Motor Shield for Arduino (DRV8825; DFRobot) stepper driver. This circuit allows the control software to operate the stepper motors in linear motion and thus control the aperture width. The aperture is set through the movement of two stimulation bars 40 × 20 mm (height, wide), built using a 3D printer Polylactic acid plastic. The position of the bars related to the subject's finger is limited in order to prevent any discomfort or injury. This is implemented by limiting the current that can be supplied to the motors. Also, the bars are built using honeycomb infill pattern of 30% density, thus, they will break if too much force is exerted against the finger. Bending will only occur if the bars are set to move beyond the distances required for testing. These two bars are located at either side of the Center-Finger Place (CFP, see Fig. 1 for details) and define the aperture width according to the size of the finger for each particular subject. In other words, testing an aperture of 1 cm would indicate the distance of 1 cm (0.5 + 0.5 cm in each side) relative to the finger of the subject, and not the actual distance between the two bars. The steppers are setup to operate at 360°/16 resolution and are connected to high precision trapezoidal spindles of 9 mm diameter, resulting in an aperture width resolution of ~0.005 mm/step. To ensure precision of the task the most critical components are the steppers, the driver, the spindle and the 3d parts (the 3d printer files are available at https://github.com/avperrotta/tactileFeedback).

Inside the box, the CFP is set to facilitate maintaining the index fingertip in a fixed position. The basis of the CFP is raised 3 cm to facilitate hand positioning with index extension. This location is monitored by a small USB camera of 640 × 480 pixel resolution that detects if the finger is properly positioned using background subtraction and frame differencing algorithms. The camera is also used to observe and record participant's finger movements. This is done through motion detection followed by synchronized time-stamped notifications to the control software. On the frontal panel of the box, there is an indicator light placed on the center of the box, right above the finger opening. This light uses a color scheme (red, yellow, green) to indicate the subject which action must be performed in each epoch of the trial (yellow – insert finger, green – maintain index finger in CFP to receive tactile stimulation or actively sample the bars, red – remove finger and push button corresponding to the aperture width: Blue – narrow and Yellow – wide). These buttons are high-quality arcade-style pushbuttons (Switch PushButton SPDT 3A 120v, SparkFun Distributor), delivering consistent and false-positive safe responses. Finally, on the left top corner of the frontal panel, a four-digit led display (14.22 mm 7-segment LED Display, 4 Digit Red Dynamic Common Cathode; S/N: 721405250844; Microtivity) can be used to transmit relevant information such as the trial number or the outcome of each trial.

The custom control software was implemented using Max programming language and was designed to provide a Graphical User Interface (GUI), where the experiment manager can calibrate the mechanics, algorithms, and experiment parameters. This software is available on Github (https://github.com/avperrotta/tactileFeedback) and is mouse-navigated and controlled. An example of the software is shown in Fig. 2. The software is set as a sequence of numbers starting in 1 and ending in 20, to facilitate the user's actions throughout the experiments. Functions associated with numbers 1–4 ensure proper communication between the Arduino and the Control computer. Functions 5–8 set the algorithm's related video capture and computer vision. Functions 9–13 relate to motor calibration. Functions 14–15 serve to add information related to the subjects gender and age. Functions 16 and 17 are related to finger size. Functions in number 18 are related to the level of difficulty to be tested in each session and with the version of the test (active: green options; or passive: blue options). It is also possible to select options that introduce visual (in the four-digit display) as well as auditory feedback after the buttons are pressed. Lastly, number 19 is related to loading the session in Arduino (19). Data is automatically saved at the end of the session.

### Behavioral protocol

**Fig. 4:**
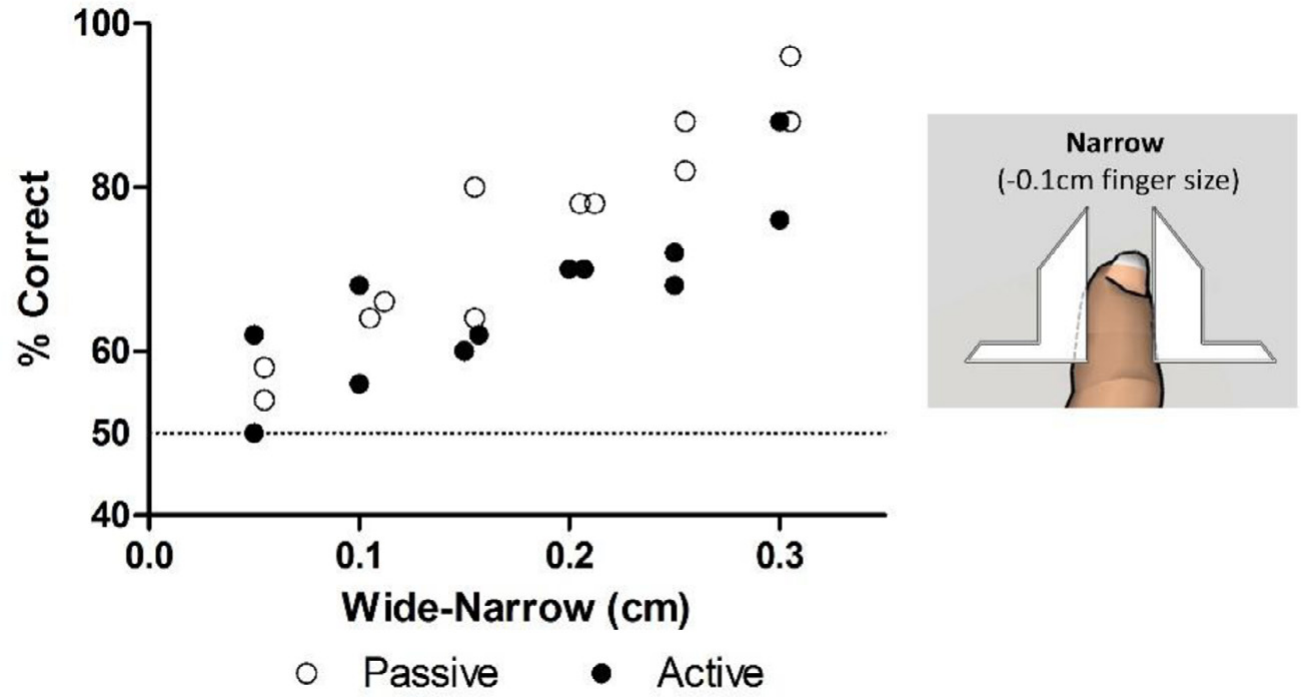
Active and passive aperture width discrimination. Behavioral performances for a single subject tested twice in each distance, in active and passive versions of the task. Each circle represents a different session. In the active version and passive versions of the task, performances dropped to near-chance values, when distances became smaller than 0.1 cm. Technical note: Circles were moved 0.05 cm in the *X* axis to prevent superposition of close values.

**Fig. 5:**
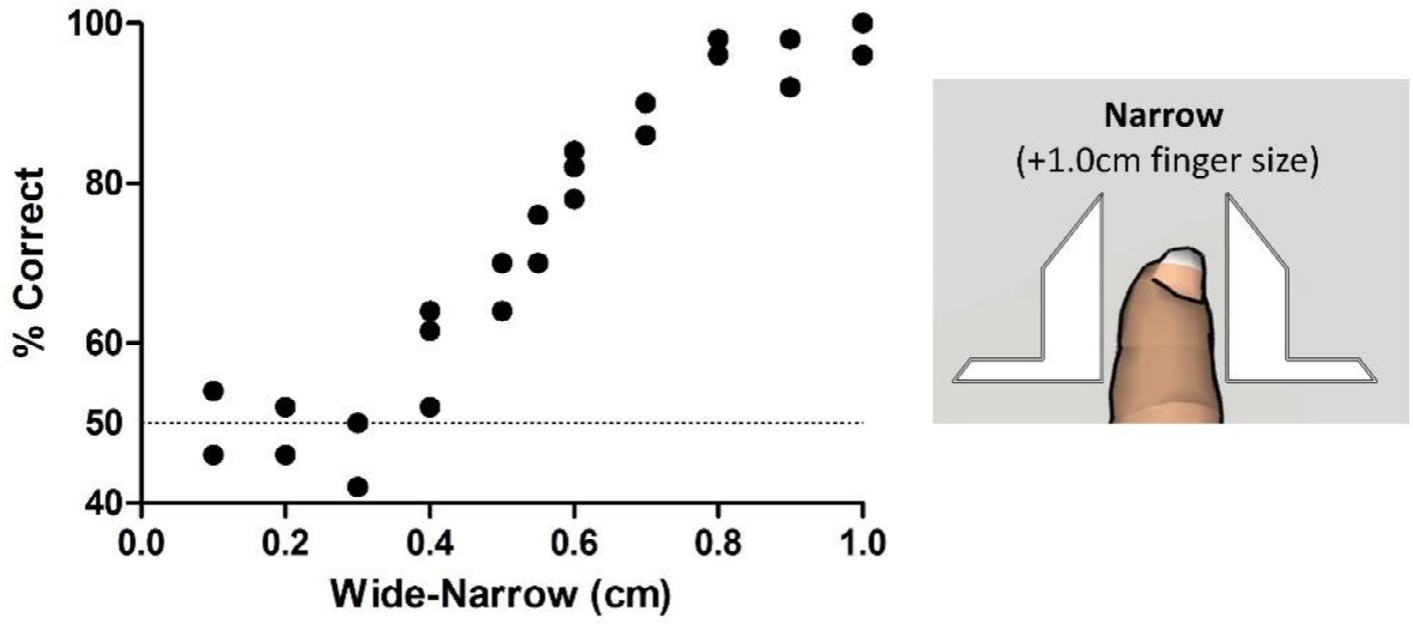
Active tactile discrimination when the narrow distance is increased. Behavioral performances of a single subject repeatedly tested in the active version of the task when the narrow distance was 1.0 cm larger than the finger size (compare to performances in Fig. 4 where narrow was −0.1 cm of the finger size). Each circle represents a different session. Performances in active tactile discrimination reached values near perfection when the narrow width was larger than the finger size (1.0 cm). For these widths, active performances dropped to near chance levels when distances became smaller than 0.5 cm.

Fig. 3 depicts the different steps of the behavioral protocol. The experimental session consisted of a total of four runs of width discrimination. Each run block was composed of 20 trials with runs alternating between passive and active tactile versions of the task. Each run contained an equal number of “wide” or “narrow” apertures trials (10 wide and 10 narrow) which were pseudo-randomized to prevent multiple consecutive trials with the same stimulus [8]. The aperture settings used in this experiment were chosen based on pilot experiments (see Fig. 4, Fig. 5). The variable-aperture width was set to one of two possible widths. In the active version, wide and narrow were set to +0.5 cm and +0.0 cm above finger size respectively. In the passive version of the task, wide and narrow were respectively set to +0.2 cm above finger size, and −0.2 cm below finger size. To calculate the finger size (step 16 of Fig. 2), the subject will leave the finger in the CFP and the experimenter will progressively move the bars until they touch the index finger on each side. This step is dependent on the subjects’ report, but can be partially controlled by the experimenter in the raw and black & white cameras. It is important to highlight here that, when the “finger size” is calculated, the subject should report feeling the bars touching and exerting some pressure on the distal interphalangeal joint. In practice, this means that the calculated finger size should actually be 1.0–2.0 mm smaller than the subject's finger size. This is of crucial importance for adequate testing during the passive version of the task [9], [15], [17]. These aperture widths however, can be adjusted when other variables of interest are studied [10], [12], [13], [14], [15], [16], [17]. For example, for studies of attention and memory these distances can be increased, decreased or made equal to allow comparing performances in active and passive versions of the task.

Each trial with a “wide” or “narrow” aperture started (i.e., stimulus onset) when the center light turned yellow, indicating that the subject had to place the finger in the CFP. Then, if the algorithm detected that the finger was in the correct position, the trial light turned green. At this point the subject was allowed to sample the aperture width for a period of time (e.g., 500 ms). Sampling the aperture width could be active or passive. If the block was active, the subject could move the finger to explore the aperture width. Otherwise, if the block was passive, the subject was required to remain with the finger in the CFP, and the bars would move towards the finger. After the discrimination, the center light turned red, and the subject was required to remove the finger from the frontal panel opening and press one of the two push buttons to indicate the aperture width of that particular trial. After a button was pressed, the four LED digital display would indicate if the response was correct or incorrect and the total number of correct responses was updated. This corresponded to the end of the trial. An intertrial interval of 500±50 ms (Mean±SEM) would then be signaled with the center light turning again red. This indicated that the subject should not introduce the finger in the frontal panel opening.

Before the EEG cap was placed, the subject was allowed a short practice session (15 trials of each condition) in which the subject was familiarized with the specific instructions to each tactile discrimination condition.

### Method validation

The present research protocol was approved by the Ethics Committee of the Universidade do Minho (SECVS 148/2016; and the Ethics Committee of the Universidade Católica Portuguesa (39/2017), according to the Code of Ethics of the World Medical Association (Declaration of Helsinki) for experiments involving humans. All participating subjects voluntarily filled an informed consent.

Due to the methodological nature of the present manuscript we will only present results from a reduced number of subjects (*n* = 9) that were tested with the goal of exploring the upper and lower bounds of the task, namely: number of trials, intertrial intervals, session duration, trial duration, active and passive discrimination limits, effects of different versions of the task in EEG recordings.

In Fig. 4, we present the behavioral results for a single subject performing the width discrimination task with multiple levels of difficulty (two sessions for each distance). In the active version of the task, the highest performances (>70%) were found for 0.3 cm. When the distances were smaller than 0.15 cm the performances in the active version of the task dropped to near chance levels. When the subject was tested in the passive version of the task, a maximum of 96% correct responses was found for distances of 0.3 cm. As the difference between the two apertures became smaller than 0.1 cm, the performance in the passive version of the task dropped to near chance levels. These results suggested that – in the present set of distances tested – the passive version of the task presented increased performances for larger distances when compared to the active version of the task. Meanwhile, both versions presented low performances when small distances were tested.

As passive width stimulation requires the bars to touch the finger, the distances tested in the passive mode must either match or be smaller than the width of the finger (otherwise, the bars will not touch the finger and the subject cannot discriminate between the two apertures). However, in active discrimination, the subject can be tested in apertures that are larger than the width of the finger.

Having this in mind, we then tested the subject in the active version of the task, but now using a different reference for the “narrow” distance. The subject was now required to explore two different apertures, but both were larger than the subject's finger. The narrow aperture was now 0.5 cm larger than the finger on both sides (an excess of 1.0 cm), and the wide aperture was varied according to the *X* axis of Fig. 5.The overall distribution of the performances now presented values that were close to chance when widths differed by 0.4 cm, and high values whenever the width difference was equal or higher than 0.6 cm (~70%). The performances reached values near 100% correct when distances were equal or above 0.8 cm. These results indicated that near perfect performances in active trials were easier to achieve if the widths tested were larger than the finger size.

**Fig. 6:**
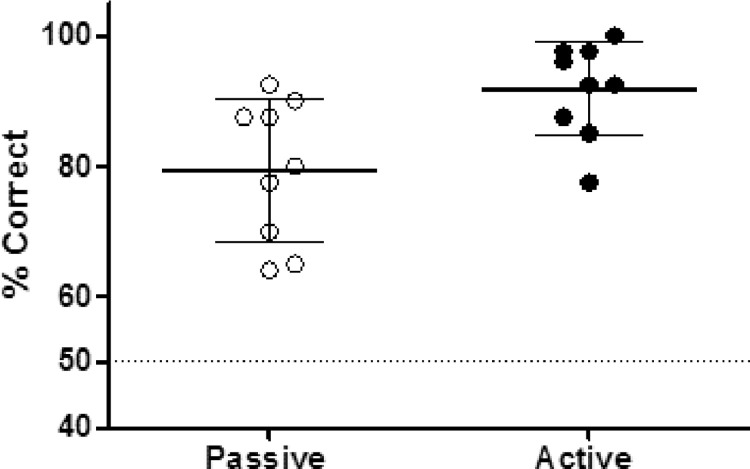
Behavioral performance in different versions of the task. A sample of nine subjects was tested in passive (empty circles) and active (filled circles) versions of the task.

Nine subjects were then tested in active and passive versions of the task according to the behavioral protocol described in Fig. 3, and the behavioral performance was analyzed. In the passive version of the task (differential width of 0.4 cm), the performance was 79.34±10.89% (mean and standard deviation) correct responses (Fig. 6, empty circles). In the active version of the task (differential width of 0.5 cm at an initial distance of 1.0 cm) the performance was 91.78±7.25% (mean and standard deviation) correct responses (Fig. 6, filled circles). These results show that a small sample of subjects was capable of performing both versions of the task with performances above chance*.*

### EEG correlates of active and passive width discrimination

Having demonstrated that subjects could present a variety of behavioral performances when tested in active or passive versions of the task, we then asked if the neurophysiological basis of each version would also be different. For this, we recorded EEG data from seven subjects performing the task in active and passive versions according to the conditions described in the behavioral protocol (Fig. 3(B)).

**Fig. 7:**
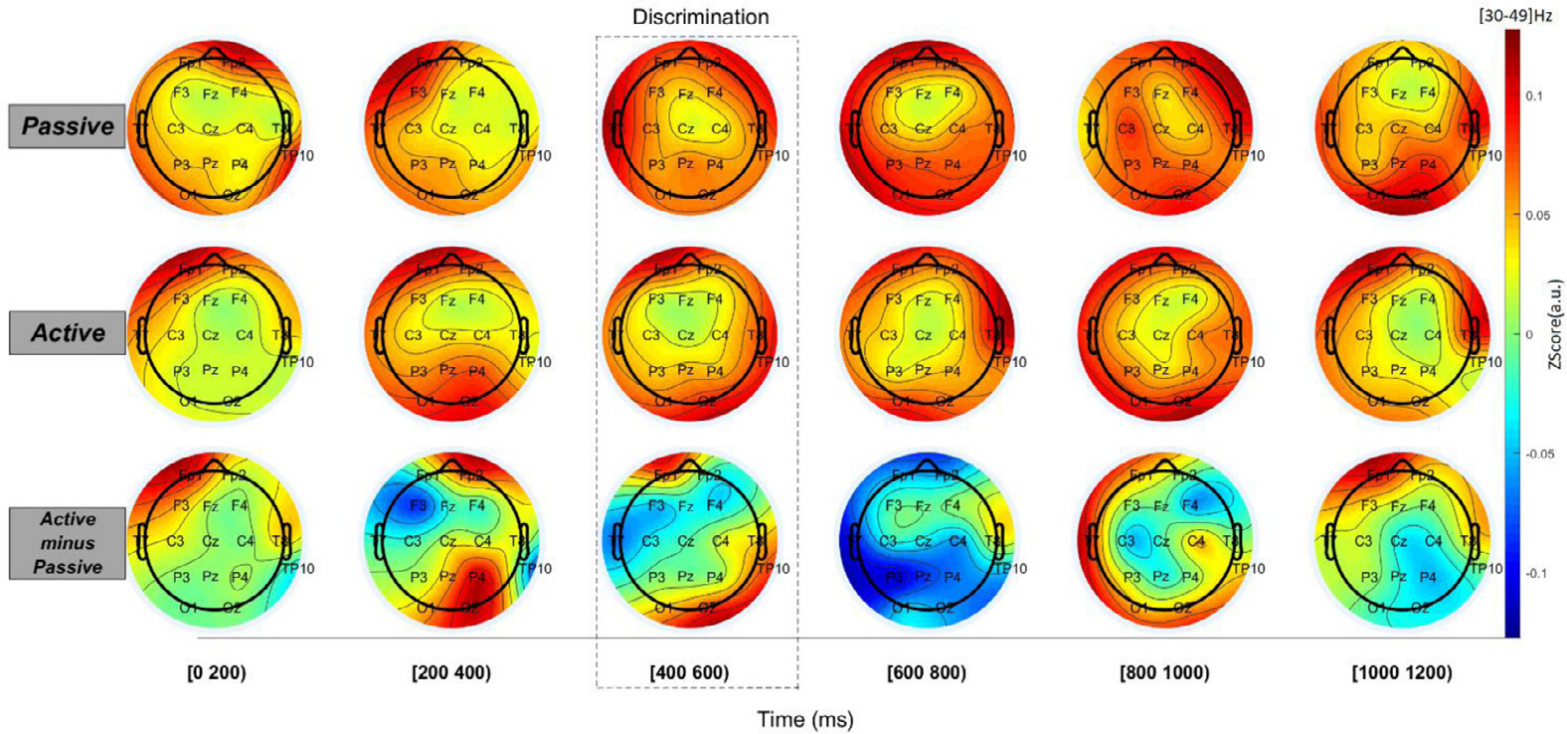
Topographical map of low gamma band EEG recordings in active and passive width discrimination. Low gamma band frequency (30–49 Hz) power of EEG recordings from seven subjects performing passive (top row) and active (middle row) versions of the task. In the bottom row the difference between topographical maps of EEG activity in the active and passive versions of the task is shown.

Analysis of EEG data of subjects performing the task revealed fundamentally different topographic maps [7], [11], [12], [13]. In Fig. 7, we present the average topographic maps resulting from EEG recordings in the active and passive versions of the task. A subset of the low gamma band frequency is presented (30–49 Hz). We have opted to show this particular range of frequencies since they are known to be associated with tactile processing in rodents [1], [2], [3] and humans [4], [18].  It should be noted however, that the stimulus sampling in active condition can also be associated with multiple other processes such as planning and execution of movement [5], [6]. For ease of presentation we have divided the trial in six different epochs of 200 ms; the Discrimination period corresponds to the (400–600)ms interval. In the passive version of the task, the topographic map showed an overall pattern of increased activity in electrodes located in the left frontal and temporal regions. Meanwhile, the active version of the task also presented an increase in activity associated with left frontal and temporal electrodes and, in addition, with the occipital lobe electrodes. These distinct patterns of EEG activity in the lower gamma frequency band (30–49 Hz) suggest that the task described here is useful to study the neural dynamics underlying active and passive tactile processing. Additional studies, specifically designed to separate other ongoing processes (e.g. planning and execution of movements), are required to properly describe the neural basis of active and passive tactile width discrimination.

Due to the methodological nature of the present manuscript, we have not analyzed here in detail the patterns of neural activity associated with other frequency bands, or task periods (e.g., pre-discrimination, response, etc.).

## Appendix Supplementary materials

Image, application 1

## References

[1] Bauer M., Oostenveld R., Peeters M., Fries P. (2006). Tactile spatial attention enhances gamma-band activity in somatosensory cortex and reduces low-frequency activity in parieto-occipital areas. J. Neurosci..

[2] Buhl E.H., Tamás G., Fisahn A. (1998). Cholinergic activation and tonic excitation induce persistent gamma oscillations in mouse somatosensory cortex in vitro. J. Physiol..

[3] Cardin J.A., Carlén M., Meletis K., Knoblich U., Zhang F., Deisseroth K., Tsai L.H., Moore C.I. (2009). Driving fast-spiking cells induces gamma rhythm and controls sensory responses. Nature.

[4] Cheng C.H., Chan P.Y.S., Niddam D.M., Tsai S.Y., Hsu S.C., Liu C.Y. (2016). Sensory gating, inhibition control and gamma oscillations in the human somatosensory cortex. Sci. Rep..

[5] Crone N.E., Miglioretti D.L., Gordon B., Sieracki J.M., Wilson M.T., Uematsu S., Lesser R.P. (1998). Functional mapping of human sensorimotor cortex with electrocorticographic spectral analysis. I. Alpha and beta event-related desynchronization. Brain: J. Neurol..

[6] Crone N.E., Miglioretti D.L., Gordon B., Lesser R.P. (1998). Functional mapping of human sensorimotor cortex with electrocorticographic spectral analysis. II. event-related synchronization in the gamma band. Brain: J. Neurol..

[7] Hyvarinen A., Karhunen J., Oja E. (2002). Independent component analysis. Stud. Inf. Control.

[8] Krupa D.J., Matell M.S., Brisben A.J., Oliveira L.M., Nicolelis M.A. (2001). Behavioral properties of the trigeminal somatosensory system in rats performing whisker-dependent tactile discriminations. J. Neurosci..

[9] Krupa D.J., Wiest M.C., Shuler M.G., Laubach M., Nicolelis M.A. (2004). Layer-specific somatosensory cortical activation during active tactile discrimination. Science.

[10] Kunicki C., Moioli R.C., Pais-Vieira M., Peres A.S.C., Morya E., Nicolelis M.A. (2019). Frequency-specific coupling in fronto-parieto-occipital cortical circuits underlie active tactile discrimination. Sci. Rep..

[11] Mahajan R., Morshed B.I. (2014). Unsupervised eye blink artifact denoising of EEG data with modified multiscale sample entropy, kurtosis, and wavelet-ICA. IEEE J. Biomed. Health Inform..

[12] Moungou A., Vezzoli E., Lombart C., Lemaire-Semail B., Thonnard J.L., Mouraux A. (2016). A novel method using EEG to characterize the cortical processes involved in active and passive touch. Proceedings of the 2016 IEEE Haptics Symposium (HAPTICS).

[13] Moungou A., Thonnard J.L., Mouraux A. (2016). EEG frequency tagging to explore the cortical activity related to the tactile exploration of natural textures. Sci. Rep..

[14] Pais-Vieira M., Lebedev M.A., Wiest M.C., Nicolelis M.A. (2013). Simultaneous top-down modulation of the primary somatosensory cortex and thalamic nuclei during active tactile discrimination. J. Neurosci..

[15] Pais-Vieira M., Kunicki C., Tseng P.H., Martin J., Lebedev M., Nicolelis M.A. (2015). Cortical and thalamic contributions to response dynamics across layers of the primary somatosensory cortex during tactile discrimination. J. Neurophysiol..

[16] Pereira A., Ribeiro S., Wiest M., Moore L.C., Pantoja J., Lin S.C., Nicolelis M.A. (2007). Processing of tactile information by the hippocampus. Proc. Natl. Acad. Sci..

[17] Thomson E., Lou J., Sylvester K., McDonough A., Tica S., Nicolelis M.A. (2014). Basal forebrain dynamics during a tactile discrimination task. J. Neurophysiol..

[18] von Lautz A.H., Herding J., Ludwig S., Nierhaus T., Maess B., Villringer A., Blankenburg F. (2017). Gamma and beta oscillations in human MEG encode the contents of vibrotactile working memory. Front. Hum. Neurosci..

