# Supplementary material for: Differential width discrimination task for active and passive tactile discrimination in humans
Source: MethodsX. 2020 Mar 19;7:100852. doi: 10.1016/j.mex.2020.100852 (PMC7155220; doi:10.1016/j.mex.2020.100852)
Supplement: Supplementary file 1 [file mmc1.docx]

**Supplementary material and Additional information:**

**Justification of method/Introduction**

Active and passive tactile processing engage fundamentally different neural network dynamics in multiple systems in rodents, and humans (Krupa et al., 2004; Pais-Vieira et al., 2013; Pais-Vieira et al., 2015; Moungou et al., 2016a, Moungou et al., 2016b; Kunicki et al., 2019). Studies using the width discrimination task for rodents (Krupa et al., 2001) have identified tactile related activity in multiple regions typically not associated with tactile processing (i.e. the somatosensory cortex), such as the primary motor cortex (Pais-Vieira et al., 2015), basal forebrain (Thomson et al., 2014), hippocampus (Pereira et al., 2007), and visual cortex (Kunicki et al., 2019). These have suggested that complex networks of cortical and subcortical regions transfer information and cooperate to allow width discrimination (Nicolelis, 1995; Nicolelis, 2011; Pais-Vieira et al., 2015; Kunicki et al., 2019). It is not known however, if the same regions are involved in width discrimination in humans. Here, we set to describe a new task for width discrimination in humans that reproduces the fundamental aspects of the rodent width discrimination task, namely the possibility of having: active and passive tactile width discrimination, reward or no-reward contingent on the correct discrimination, as well as the ability to set multiple levels of difficulty. Lastly, to determine if the present task generated different neurophysiological correlates for active and passive stimulation, EEG data were also recorded while subjects performed the task.

**EEG recording and data analysis**

EEG activity was recorded using the Brain Vision Recorder Software (version 1.22.0001, Brain Products GmbH, Germany). The recording was taken from 16 equidistant scalp sites mounted in a cap system. A low-density EEG of 16 electrodes (sintered Ag/AgCl ring electrodes) was used to require less time to set up the electrode cap. EEG gel-based electrodes were referenced to FCz for impedance calculation and placed on the scalp according to the international 10–20 system. For signal processing each electrode was referenced to the average of all trials recorded. The impedance of all electrode channels was held below 50 kΩ. The signals were amplified and then sampled at rate of 500Hz. The participant was instructed about the experimental task and procedure of the experiment to avoid irrelevant movements, eye blinks/movements, and scratching during recordings.

Data were processed offline with EEGLAB (v.2019.0) using Matlab (version 2018b) platform. Primarily, a Notch filter was applied to remove the harmonics of power line at 50 Hz, followed by re-referencing to an average reference, on continuous datasets to obtain zero-mean datasets for Independent Component Analysis (ICA) (Hyvarinen et al., 2002). Each continuous dataset was composed of multiple trials, and each trial was composed of multiple epochs. A total of six different 200ms epochs are presented here, even though trials could last from 2 to 10 seconds (since some subjects could take longer to make a response). Here we have only focused our analysis in the discrimination period, which is centered at 400-600ms and was kept constant in all trials. In other words, our analysis is centered in the period that was similar for all trials and all subjects.

Lastly, after the above preprocessing, ICA decomposition was done, and eye-blink related sources were removed using kurtosis (Mahajan and Morshed, 2014). Also, visual inspection of Event Related Potentials (ERPs) was performed to ensure that clean recordings were obtained and no artifacts were present.

**Additional notes on particulars of the behavioral task and sessions**

In addition to the results described above, two additional subjects performed other versions of the task to allow describing general parameters such as the number of trials, intertrial interval, and session duration. These subjects reported that performing 100 trials in the passive version of the task was too exhausting. According to subjects’ reports, increased engagement during the passive version of the task could be achieved when one or more of the following were present: 1) reducing the number of trials in a session; 2) starting the session with the active version of the task, allowing the subject to rest for 5 minutes, and then performing the passive version of the task; 3) performing the task without the EEG recording (which increased the overall testing time as well as discomfort). Besides, subjects reported that the active version of the task was more interesting than the passive version.

We have not observed a clear effect of learning when the same subject was tested twice in the task. However, we have found that subjects usually required an initial period of 10-15 trials to get used to the sequence of actions necessary to accurately perform the task (i.e. wait for the green light, remove finger at red light, choose button corresponding to the correct width). Also, when the initial instructions were given to the participant, a short period of tactile and visual exploration of the aperture bars and the behavioral apparatus was allowed (~1 minute). This period included opening the top of the box to allow the subject visual and tactile inspection of the bars and CPF.
